# Influence of surgical evacuation on pregnancy outcomes of subsequent embryo transfer cycle following miscarriage in an initial IVF cycle: a retrospective cohort study

**DOI:** 10.1186/s12884-019-2543-9

**Published:** 2019-11-08

**Authors:** Junan Meng, Mengchen Zhu, Wenjuan Shen, Xiaomin Huang, Haixiang Sun, Jianjun Zhou

**Affiliations:** 1 Reproductive Medicine Center, The Affiliated Drum Tower Hospital of Nanjing University Medical School, Zhongshan Road 321#, Nanjing, 210008 People’s Republic of China; 2 Department of Obstetrics and Gynecology, Suqian People’s Hospital of Drum Tower Hospital Group, Suqian, 223800 China

**Keywords:** Embryo transfer, Miscarriage, Surgical evacuation, Pregnancy outcomes, Endometrial thickness

## Abstract

**Background:**

It is still uncertain whether surgical evacuation adversely affects subsequent embryo transfer. The present study aims to assess the influence of surgical evacuation on the pregnancy outcomes of subsequent embryo transfer cycle following first trimester miscarriage in an initial in vitro fertilization and embryo transfer (IVF-ET) cycle.

**Methods:**

A total of 645 patients who underwent their first trimester miscarriage in an initial IVF cycle between January 2013 and May 2016 in Nanjing Drum Tower Hospital were enrolled. Surgical evacuation was performed when the products of conception were retained more than 8 h after medical evacuation. Characteristics and pregnancy outcomes were compared between surgical evacuation patients and no surgical evacuation patients. The pregnancy outcomes following surgical evacuation were further compared between patients with ≥ 8 mm or < 8 mm endometrial thickness (EMT), and with the different EMT changes.

**Results:**

The EMT in the subsequent embryo transfer cycle of surgical evacuation group was much thinner when compared with that in the no surgical evacuation group (9.0 ± 1.6 mm vs. 9.4 ± 1.9 mm, *P* = 0.01). There was no significant difference in implantation rate, clinical pregnancy rate, live birth rate or miscarriage rate between surgical evacuation group and no surgical evacuation group (*P* > 0.05). The live birth rate was higher in EMT ≥ 8 mm group when compared to < 8 mm group in surgical evacuation patients (43.0% vs. 17.4%, *P* < 0.05).

**Conclusions:**

There was no significant difference in the pregnancy outcomes of subsequent embryo transfer cycle between surgical evacuation patients and no surgical evacuation patients. Surgical evacuation led to the decrease of EMT, especially when the EMT < 8 mm was association with a lower live birth rate.

## Background

Miscarriage is defined as a spontaneous loss of an intrauterine pregnancy before the fetus is able to survive independently. First trimester miscarriage is defined as ≤ 12 weeks gestation of miscarriage [1, 2]. The occurrence of miscarriage was 10–20% in natural pregnant women, with 80% of pregnancy losses occurring in the first trimester [3]. Studies had shown that the rate of miscarriage among in vitro fertilization and embryo transfer (IVF-ET) pregnant women was similar to that of natural pregnant women [4]. A study showed that the miscarriage rate was 14.7% in 62,228 clinical pregnancies after IVF treatment in 1996–1998 in USA [5], which decreased to 9.5% in 2015 [6]. A retrospective cohort study of 112,549 IVF cycles during 1999–2008 in the UK showed the miscarriage rate was 8.3% [7].

Studies showed that miscarriage might have adverse effects on subsequent pregnancy [8, 9]. The outcome of an initial pregnancy seemed to have an important influence on the whole subsequent reproductive outcomes [8]. The treatment methods of miscarriage may also impact subsequent pregnancies. The managements of miscarriage usually contain expectant management, medical evacuation and surgical evacuation. For many years, the standard management of early pregnancy loss was dilatation and curettage (D&C) [10]. In recent years, vacuum aspiration or suction curettage had also been commonly used in miscarriage management [11]. It is difficult to fully distinguish these techniques in clinical practice, as curettage and aspiration techniques are commonly used in conjunction. Surgical evacuation may associated with complications such as bleeding, infection, endometrium trauma, intrauterine adhesions, scarring and perforation of the uterus [12–15]. A study reported that surgical evacuation could lead to reduction of endometrium [13]. And there were some studies showed that endometrium thickness was associated with pregnancy outcomes [16, 17]. However, the influence of surgical evacuation on pregnancy outcome remains controversial [18, 19].

IVF patients who desired a baby have described anxiety of whether the experience of miscarriage was association with the subsequent pregnancy outcomes [20]. There have been few previous studies that have investigated the influence of miscarriage and surgical evacuation after assisted reproduction technology (ART) treatment. Some studies showed the influence of a previous miscarriage on subsequent embryo transplantation, but the role of surgical evacuation was not explored [21–23]. It was found that surgical evacuation produced significantly adverse effects on the reproductive outcomes of subsequent frozen embryo transfer (FET) treatment within six-month, while the influence on reproductive outcomes was not significant longer than six-month [24]. However, it was concluded that surgical evacuation had no influence on subsequent IVF pregnancy outcomes when used as a treatment for miscarriage prior to an IVF cycle [25]. Whether surgical evacuation has adverse effect on subsequent embryo transfer is still uncertain.

The present study aims to explore the pregnancy outcomes and the role of surgical evacuation in subsequent embryo transfer cycle in patients who have experienced miscarriage in their first embryo transfer treatment.

## Methods

### Study population

The patients who under their first trimester miscarriage in an embryo transfer cycle between January 2013 and May 2016 in Nanjing Drum Tower Hospital were included in this retrospective cohort study. First trimester miscarriage was defined as a pregnancy loss before 12 completed weeks of gestation [26], including embryonic or fetal death, anembryonic gestation, and spontaneous miscarriage. Miscarriage patients with types of embryonic or fetal death or anembryonic gestation received medical evacuation as initial treatment. Vacuum aspiration was used as the surgical evacuation method to expel the residual pregnancy products when they retained in uterus more than 8 h after medical evacuation. The exclusion criteria were patients who used donor eggs or sperm, had a previous surgical evacuation treatment, had no subsequent embryo transfer cycle, or were diagnosed with adenomyosis, congenital uterine cavity anomalies, or chromosome abnormalities. All data in this retrospective cohort study were obtained from routine outpatient testing and previous follow-up medical records. This retrospective cohort study obtained ethics board approval from the Reproductive Medical Ethics Committee of Nanjing Drum Tower Hospital.

### Clinical characteristics

Basic characteristics such as age, body mass index (BMI), antral follicle count (AFC), basal serum levels of follicle-stimulating hormone (FSH), luteinizing hormone (LH), oestradiol (E2) and progesterone (P) were collected in all patients.

The IVF outcomes included the number of retrieved oocytes, the number of transferred embryos, treatment methods, endometrial thickness (EMT) and top quality embryos rate. EMT was measured on the day of hCG (human chorionic gonadotropin) injection (hCG day) in fresh embryo transfer cycle or on the day of endometrium endocrine transformation in frozen-thawed embryo transfer cycle by transvaginal ultrasonography. Top quality embryos were considered to be eight to ten cells of cleavage-stage embryo, even size, and less than 15% fragmentation. For blastocyst embryos, top quality embryos referred that embryos which were graded ≥ BB level according to Gardner’s inner cell mass and trophectoderm morphology grading [27].

The miscarriage characteristics included gestational age before miscarriage, EMT change, and times of cycle cancellation and interval time between two cycles. The interval time between two cycles was defined as the number of days between the date of embryo transfer in first cycle and the date of that in second cycle. EMT change was defined as the reduction of EMT in the second cycle compared to that in the first cycle.

### Groups for investigation

The cohort was divided into two groups according to surgical evacuation or no surgical evacuation. Surgical evacuation or no surgical evacuation patients with 0, 1 or 2 top embryos transferred were grouped respectively. Depending on the EMT change, the surgical evacuation group was further divided quarterly into four groups by the 25th, 50th, and 75th percentile among patients respectively: group 1 (< 0 mm); group 2 (≥ 0 mm and < 1 mm); group 3 (≥ 1 mm and < 2 mm); group 4 (≥ 2 mm). To further explore the influence of thin endometrium, cycles following surgical evacuation were divided according to EMT with 8 mm cut-off values into two groups (< 8 mm and ≥ 8 mm).

### Outcomes statistics

The main outcomes were implantation rate, clinical pregnancy rate, live birth rate and first trimester miscarriage rate in the second cycle. Clinical pregnancy was defined as the demonstration of intrauterine gestational sac and fetal cardiac activity 4 weeks after embryo transfer the demonstrated by ultrasonography. Live birth was defined as the delivery of a living baby after 28 weeks of gestation. Clinical pregnancy rate and live birth rate was respectively calculated by the ratio of patients that had clinical pregnancy or live birth in total patients included. Miscarriage rate was calculated by the ratio of patients that underwent miscarriages in total clinical pregnancy patients.

### Statistical analysis

IBM SPSS v20.0 was applied in data statistical analysis. Measurement data were calculated by mean ± standard deviation. To analyze differences in clinical characteristics which fitted normal distribution among the study groups, Independent-samples Student’s t-test or one-way ANOVA was used. The non-normal distribution data were conducted with the Kruskal-Wallis Test. Categorical data were calculated by percentages, and were compared by Chi-squared test or Fisher’s exact test. *P* < 0.05 was considered to be statistically significant.

## Results

### Baseline clinical characteristics

**Fig. 1:**
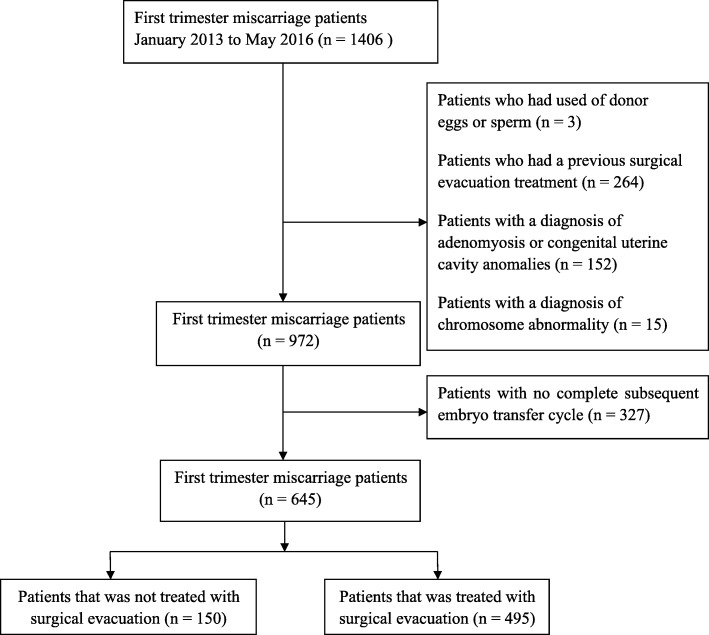
Flowchart of study patients

A total of 645 patients were included in the study, aged 32.0 ± 4.8 years old, with 346 IVF/ICSI cycles (53.6%) and 299 FET cycles (46.4%). The exclusion and the selection procedure were illustrated in Fig. 1. The data of main clinical characteristics were demonstrated in Table 1. The mean EMT on hCG day was 10.0 ± 1.9 mm in first cycle and 9.3 ± 1.5 mm in second cycle respectively, which decreased 0.7 ± 1.8 mm following once miscarriage. Four hundred ninety-five patients underwent surgical evacuation whereas 150 patients did not undergo surgical evacuation. In subsequent cycle, the overall implantation rate was 40.8% (462/1133), the clinical pregnancy rate was 54.7% (353/645), the live birth rate was 42.8% (276/645) and the miscarriage rate was 16.4% (58/353). There were 80.1% (521/645) miscarriage patients as a result of embryonic or fetal death, while 11.5% (74/645) of spontaneous miscarriage occurred before ultrasound diagnosis, and 7.9% (51/645) during anembryonic gestation.

**Table 1:** Basic clinical characteristics and pregnancy outcomes of subsequent embryo transfer cycle following miscarriage in an initial IVF cycle

Variables	N (mean ± SD)
Number	645
Age (year)	32.0 ± 4.8
BMI (kg/m^2^)	22.6 ± 3.3
AFC (n)	14.5 ± 5.8
Baseline FSH (IU/L)	7.4 ± 2.3
Baseline LH (IU/L)	4.6 ± 3.4
E2 on hCG day (pg/mL)	4714.2 ± 2638.1
P on hCG day (ng/mL)	1.0 ± 0.9
Gestational age of miscarriage (week)	9.1 ± 2.2
Types of miscarriage in first cycle	
Embryonic or fetal death	521
Spontaneous miscarriage	74
Anembryonic gestation	51
Interval time between two cycles (day)	367.3 ± 187.9
Oocytes retrieved in first cycle (n)	5.8 ± 6.1
Number of embryos transferred in first cycle (n)	1.9 ± 0.3
Number of embryos transferred in second cycle (n)	1.8 ± 0.4
Treatment methods in first cycle (n)	
IVF/ICSI	346
FET	299
Treatment methods in second cycle (n)	
IVF/ICSI	60
FET	585
Endometrial thickness in first cycle (mm)	10.0 ± 1.9
Endometrial thickness in second cycle (mm)	9.3 ± 1.5
Endometrial thickness change (mm)	0.7 ± 1.8
Take surgical evacuation after miscarriage (n)	495
Non surgical evacuation after miscarriage (n)	150
Implantation rate in second cycle (%)	40.8 (462/1133)
Clinical pregnancy rate in second cycle (%)	54.7 (353/645)
Live birth rate in second cycle (%)	42.8 (276/645)
Miscarriage rate in second cycle (%)	16.4 (58/353)

*Abbreviations*: *BMI* body mass index, *AFC* antral follicle count, *FSH* follicle-stimulating hormone, *LH* luteinizing hormone, *E2* oestradiol, *P* progesterone, *hCG* human chorionic gonadotropin, *IVF* in vitro fertilization and embryo transfer, *ICSI* intracytoplasmic sperm injection, *FET* frozen embryo transfer

### Outcome differences between surgical evacuation and control groups

EMT in the subsequent cycles in the surgical evacuation group was much thinner when compared to that in the no surgical evacuation group (9.0 ± 1.6 mm vs. 9.4 ± 1.9 mm, *P* = 0.01) (Table 2). The change of EMT after miscarriage in the surgical evacuation group was significantly more than that in no surgical evacuation group (0.8 ± 1.8 mm vs. 0.4 ± 1.6 mm, *P* < 0.001). The surgical evacuation group had longer interval time between two cycles than the no surgical evacuation group (387.3 ± 192.2 day vs. 300.0 ± 157.9 day, *P* < 0.001). There was no significant difference in implantation rate (41.3% vs. 39.1%, *P* > 0.05), clinical pregnancy rate (54.6% vs. 55.3%, *P* > 0.05), live birth rate (41.8% vs. 46.0%, *P* > 0.05) or miscarriage rate (17.4% vs. 13.3%, *P* > 0.05) between these two groups.

**Table 2:** Clinical characters and pregnancy outcomes in subsequent embryo transfer cycle of no surgical evacuation and surgical evacuation patients

	No surgical evacuation	Surgical evacuation	*P* value
Number	150	495	–
Age (year)	32.1 ± 5.0	32.0 ± 4.8	0.85
BMI (kg/m^2^)	22.4 ± 3.2	22.6 ± 3.3	0.48
AFC (n)	14.4 ± 5.8	14.5 ± 5.8	0.95
Baseline FSH (IU/L)	7.3 ± 2.0	7.4 ± 2.4	0.58
Baseline LH (IU/L)	4.6 ± 3.7	4.6 ± 3.3	0.98
E2 on HCG day (pg/mL)	4510.2 ± 2613.1	4776.3 ± 2645.2	0.28
P on HCG day (ng/mL)	1.0 ± 1.2	1.0 ± 0.8	0.69
gestational age of miscarriage (week)	8.5 ± 1.9	9.3 ± 2.3	< 0.001
Interval time between two cycles (day)	300.0 ± 157.9	387.3 ± 192.2	< 0.001
Times of cycle cancellation before second cycle (n)	0.3 ± 0.8	0.4 ± 0.7	0.86
Endometrial thickness in first cycle (mm)	10.0 ± 1.7	10.0 ± 1.9	0.86
Endometrial thickness in second cycle (mm)	9.5 ± 1.9	9.0 ± 1.6	0.01
Endometrial thickness change (mm)	0.4 ± 1.6	0.8 ± 1.8	< 0.001
Number of embryos transferred in first cycle (n)	1.9 ± 0.3	1.9 ± 0.3	0.67
Number of embryos transferred in second cycle (n)	1.7 ± 0.5	1.8 ± 0.4	0.50
Number of top quality embryos in second cycle (n)	0.9 ± 0.8	1.1 ± 0.8	0.03
Rate of blastocyst embryo in second cycle (%)	26.7 (40/150)	24.6 (122/495)	0.59
Rate of endometrial thickness < 8 mm in second cycle (%)	18.7 (28/150)	17.2 (85/495)	0.67
Implantation rate in second cycle (%)	39.1 (102/261)	41.3 (360/872)	0.53
Clinical pregnancy rate in second cycle (%)	55.3 (83/150)	54.6 (270/495)	0.87
Live birth rate in second cycle (%)	46.0 (69/150)	41.8 (207/495)	0.36
Miscarriage rate in second cycle (%)	13.3 (11/83)	17.4 (47/270)	0.37

The surgical evacuation group had a higher number of top quality embryos in the second cycle than the no surgical evacuation group (1.1 ± 0.8 vs. 0.9 ± 0.8, *P* = 0.03). We further explored the association with 0, 1 or 2 top embryos transferred numbers and pregnancy outcomes in subsequent embryo transfer cycle with Chi-squared test. There was no significant difference in pregnancy outcomes in subsequent embryo transfer cycle between no surgical evacuation and surgical evacuation patients with 0, 1 or 2 top embryos transferred (*P* > 0.05) (Table 3).

**Table 3:** Pregnancy outcomes in subsequent embryo transfer cycle with 0, 1 or 2 of top quality embryos transferred in no surgical evacuation and surgical evacuation patients

Number of Top embryos		No surgical evacuation	Surgical evacuation	*P* value
0	Number	55	144	–
	Implantation rate (%)	35.2 (32/91)	37.5 (84/224)	0.70
	Clinical pregnancy rate (%)	43.6 (24/55)	47.9 (69/144)	0.59
	Live birth rate (%)	40.0 (22/55)	37.5 (54/144)	0.75
	Miscarriage rate (%)	8.3 (2/24)	17.4 (12/69)	0.46
1	Number	61	167	–
	Implantation rate (%)	42.2 (43/102)	41.8 (117/280)	0.95
	Clinical pregnancy rate (%)	60.7 (37/61)	55.7 (93/167)	0.50
	Live birth rate (%)	44.3 (27/61)	41.9 (70/167)	0.75
	Miscarriage rate (%)	21.6 (8/37)	20.4 (19/93)	0.88
2	Number	34	184	–
	Implantation rate (%)	39.7 (27/68)	43.2 (159/368)	0.59
	Clinical pregnancy rate (%)	64.7 (22/34)	59.8 (110/184)	0.59
	Live birth rate (%)	58.8 (20/34)	45.1 (83/184)	0.14
	Miscarriage rate (%)	4.6 (1/22)	14.6 (16/110)	0.20

### Pregnancy outcomes of subsequent embryo transfer cycle between EMT < 8 mm group and EMT ≥ 8 mm group following surgical evacuation

The live birth rate in the second cycle following surgical evacuation was higher in EMT ≥ 8 mm group when compared to EMT < 8 mm group (43.0% vs. 17.4%, *P* = 0.02) (Table 4). EMT < 8 mm group had a longer interval time between the two cycles than EMT ≥ 8 mm group (473.3 ± 228.5 day vs. 377.4 ± 193.7 day, *P* = 0.02) and had a higher number of cycle cancellation times in subsequent cycle (1.1 ± 1.4 vs. 0.3 ± 0.6, *P* < 0.001).

**Table 4:** Clinical characters and pregnancy outcomes of patients with < 8 mm or ≥ 8 mm endometrial thickness in subsequent embryo transfer cycle following surgical evacuation

	< 8 mm	≥ 8 mm	*P* value
Number	23	472	
Age (year)	31.6 ± 4.1	32.0 ± 4.8	0.52
BMI (kg/m^2^)	22.5 ± 2.8	22.7 ± 3.3	0.80
AFC (n)	14.7 ± 6.0	14.4 ± 5.7	0.58
Baseline FSH (IU/L)	7.1 ± 1.8	7.4 ± 2.4	0.57
Baseline LH (IU/L)	5.6 ± 4.3	4.9 ± 8.8	0.72
E2 on HCG day (pg/mL)	5405.9 ± 2483.9	4745.5 ± 2651.4	0.24
P on HCG day (ng/mL)	1.4 ± 0.9	1.0 ± 0.8	0.02
Endometrial thickness in first cycle (mm)	8.3 ± 1.0	10.1 ± 1.9	< 0.001
Endometrial thickness in second cycle (mm)	6.7 ± 0.3	9.3 ± 1.4	< 0.001
Endometrial thickness change (mm)	1.5 ± 1.0	0.8 ± 1.8	< 0.001
Gestational age of miscarriage (week)	9.5 ± 1.5	9.3 ± 2.3	0.65
Interval time between two cycles (day)	473.3 ± 228.5	377.4 ± 193.7	0.02
Times of cycle cancellation in second cycle (n)	1.1 ± 1.4	0.3 ± 0.6	< 0.001
Number of top quality embryos in second cycle (n)	1.4 ± 0.8	1.1 ± 0.8	0.06
Number of embryos transferred in second cycle (n)	1.9 ± 0.3	1.8 ± 0.4	0.14
Implantation rate in second cycle (%)	27.9 (12/43)	42.0 (348/829)	0.07
Clinical pregnancy rate in second cycle (%)	39.1 (9/23)	55.7 (263/472)	0.11
Live birth rate in second cycle (%)	17.4 (4/23)	43.0 (203/472)	0.02
Miscarriage rate in second cycle (%)	44.4 (4/9)	16.4 (43/263)	0.08

### EMT change and pregnancy outcomes in subsequent embryo transfer cycle following surgical evacuation

Table 5 showed the pregnancy outcomes between four groups categorized by EMT change, which was represented the reduction of EMT in second cycle compared to that in the first cycle, had no statistically significant differences (*P* > 0.05) in patients following surgical evacuation. With the increase of EMT change (< 0 mm vs. 0-1 mm vs. 1–2 mm vs. ≥ 2 mm), the number of cycle cancellation times increases (0.1 ± 0.5 vs. 0.2 ± 0.5 vs. 0.5 ± 0.8 vs. 0.5 ± 0.9, *P* < 0.001). The EMT in subsequent cycle was thinner in 1–2 mm or ≥ 2 mm groups than that in < 0 mm or 0–1 mm groups (*P* < 0.001).

**Table 5:** Clinical characters and pregnancy outcomes of patients with different EMT change in subsequent embryo transfer cycle following surgical evacuation

	< 0 mm (1)	0-1 mm (2)	1–2 mm (3)	≥ 2 mm (4)	*P* value
Number	111	133	149	102	–
Age (year)	31.2 ± 4.5	32.4 ± 4.9	32.5 ± 5.1	31.5 ± 4.3	0.07
BMI (kg/m^2^)	23.2 ± 3.5	22.3 ± 3.20	22.6 ± 3.2	22.5 ± 3.2	0.20
Baseline FSH (IU/L)	7.0 ± 2.8	7.4 ± 2.3	7.5 ± 2.5	7.6 ± 1.7	0.31
E2 on HCG day (pg/mL)	5146.5 ± 3128.9	4951.9 ± 2665.5	4536.1 ± 2373.4	4502.6 ± 2392.9	0.17
P on HCG day (ng/mL)	0.9 ± 0.5	1.1 ± 1.0	1.0 ± 0.9	0.9 ± 0.6	0.35
Gestational age of miscarriage (week)	9.21 ± 2.24	9.37 ± 2.30	8.97 ± 1.43	9.64 ± 3.15	0.14
Interval time between two cycles (day)	389.0 ± 181.8	390.3 ± 182.7	375.2 ± 204.4	403.2 ± 195.4	0.73
Times of cycle cancellation in second cycle (n)	0.1 ± 0.5	0.2 ± 0.5	0.5 ± 0.8	0.5 ± 0.9	< 0.001
Number of embryos transferred in second cycle (n)	1.8 ± 0.4	1.7 ± 0.5	1.8 ± 0.4	1.8 ± 0.4	0.42
Number of top quality embryo in second cycle (n)	1.2 ± 0.8	1.1 ± 0.8	1.1 ± 0.9	1.0 ± 0.4	0.45
Endometrial thickness in second cycle (mm)	10.2 ± 1.8	9.0 ± 1.2	8.5 ± 1.4	8.6 ± 1.6	< 0.001
Implantation rate in second cycle (%)	44.3 (89/201)	41.1 (94/229)	38.7 (101/261)	42.0 (76/181)	0.61
Clinical pregnancy rate in second cycle (%)	59.5 (66/111)	53.4 (71/133)	50.3 (75/149)	58.8 (60/102)	0.40
Live birth rate in second cycle (%)	52.3 (58/111)	44.4 (59/133)	40.9 (61/149)	40.2 (41/102)	0.24
Miscarriage rate in second cycle (%)	21.2 (14/66)	19.7 (14/71)	12.0 (9/75)	16.7 (10/60)	0.47

## Discussion

When a pregnancy ends in first trimester miscarriage, the common treatments are expectant management, medical evacuation or surgical evacuation. Surgical evacuation remains a common treatment for miscarriage when other methods fail. There were 76.7% patients in present study received surgical evacuation. Such a high surgical evacuation rate may be due to embryo developmental arrest in IVF. Embryo developmental arrest typically manifests as embryonic death or intrauterine fetal death on ultrasound, which results in delayed miscarriage due to the difficulty of completely expelling the products of conception, while about four-fifths miscarriage patients with embryonic or fetal death in present study. According to the guidelines (WHO Clinical Practice Handbook for Safe Abortion; ACOG guideline) [28, 29], surgical evacuation is warranted when women have heavy bleeding, fever or retained products of conception beyond 3–4 h. A retrospective review showed that 92.5% miscarriage patients chose surgical evacuation in hospital treatment and 51% in family physicians treatment in Canada [30]. It was considered in a study that the surgical evacuation was the standard management of early pregnancy failure, and surgical curettage had great advantages in complete evacuation rate and the bleeding time [31]. Some clinicians proposed surgical evacuation as an effective method that prevented prolonged course to avoid the negative emotion of patients [32].

Our results have shown that women who underwent surgical evacuation had thinner EMT than those who did not. Although it had a wide range of applications, the surgical evacuation, which could lead to the altering secretion of inflammatory cytokines and the thinning of endometrium, was usually considered as a damage factor of endometrium in the previous study [18]. A retrospective study found that surgical evacuation was revealed as a cause for endometrial thinning in miscarriage [13]. It was found that iatrogenic damage such as repeated or vigorous curettage might be the reason for thin endometrium and repeated surgical evacuation wound played an increasingly important role in gradual endometrial thinning [13, 33]. Based on these studies, it could be considered that surgical evacuation has a direct association with EMT reduction.

It was shown the lower live birth rate when EMT thinner than 8 mm in the surgical evacuation group, but we also found that there was no association between the rate of live birth and the degree of endometrial changes. We considered that the live birth rate was significantly decreased only when the endometrium was thinner than 8 mm, not depend on the value of EMT change. The clinical significance of EMT observed among ART patients has remained controversial. A differing opinion concludes thinner or thicker endometrium has no significant influence on pregnancy outcomes [34]. A meta-analysis showed that there was no difference in the average EMT (ranged from 7.7 ± 3.5 mm to 12.1 ± 2.6 mm) between pregnant women and no pregnant woman (OR: 0.51, 95% CI: − 0.05, 1.07) [34]. However, some studies reported that a lower live birth rate was found in patients with a relatively thinner endometrium [33, 35], the live birth rate was 15.6% with 5 mm or less endometrial thickness and gradually increased to 33.1% with an endometrial thickness of 10 mm, live birth rate was positive linearly with EMT [33]. The live birth rate was reported higher in a relatively thicker endometrium, which was 30.38, 5.73, and 54.55% respectively in patients with EMT ≤ 8 mm, 9–14 mm and ≥ 15 mm [35]. In addition, one review has shown a similar result of the present study, in which the live birth rate exhibited decreasing trends only when the EMT thinner than 7 mm [36]. The pathophysiology of a decrease in the live birth rate due to thin endometrium is not certain. It was suggested that the reason that thin endometrium patients had difficulty in implantation might be associated with oxygen tension and insufficient angiogenesis [37].

As a treatment for miscarriage, one instance of surgical evacuation showed no significant influence on pregnancy outcome in the subsequent embryo transfer cycle in our study. In the present study, patients underwent one time of surgical evacuation and had an average EMT of 9.0 ± 1.6 mm, which reached the recommended 9 mm EMT that could ensure a satisfying pregnancy rate [38]. Even in the EMT group that decreased more than 2 mm, the average EMT was thicker than 8 mm in the subsequent cycles. It seems to explain to some extent why the pregnancy outcomes of surgical evacuation patients had not been significantly affected. As the previous study suggested that live birth rate has no significant change when the EMT thicker than 8 mm [36].

Several studies of natural pregnant women came to a similar conclusion [19, 39]. A study reported that medical or surgical evacuation showed no difference in the conception rate and pregnancy outcome in long-term following miscarriage [19]. Another study suggested that the pregnancy outcomes and miscarriage rate in subsequent pregnancy were similar between curettage versus nonsurgical management in early miscarriage [39]. In a previous study that related to EMT and pregnancy outcome, EMT was temporarily briefly reduced and had no significant effect on live birth rate following surgical evacuation [40]. In a systematic review, undifferentiated live birth rate and miscarriage rate were reported in surgical evacuation patients and expectantly patients [41].

Some patients had one or more occurrences of cycle cancellation before a subsequent complete cycle. Cycle cancellation was usually due to thin endometrium to get a thicker endometrium for embryo transfer. The frequency of cycle cancellations increased with the decrease of the EMT. When EMT change was < 0 mm, 0-1 mm, 1–2 mm or ≥ 2 mm, the cycle cancellations times was 0.1 ± 0.5, 0.2 ± 0.5, 0.5 ± 0.8 or 0.5 ± 0.9 respectively. This difference in cancellation times was even more pronounced in thin endometrium patients. More than one instance of surgical evacuation could possess more significantly negative influences on subsequent pregnancy outcomes [13]. Unnecessary surgical evacuation should be avoided since it has an effect on EMT, and thin endometrium has shown to be associated with a lower live birth rate. Certainly, patients who underwent incomplete miscarriage that could combine with excessive bleeding, unstable vital signs, obvious signs of infection should be performed surgical evacuation immediately [42].

Compared to women who conceived naturally, infertile women conceived by ART had greater psychological pressure, which made the trend to choose less invasive intervention and avoid surgical complications for them [43]. Since the infertility women conceived by ART are actively seeking to conceive again following their miscarriages, unnecessary surgical evacuation which could reduce the EMT and even the reproductive success of future live birth should be avoided when possible. Our results suggested that one surgical evacuation procedure did not affect the pregnancy outcome of subsequent cycles, which might help doctors consult with patients in case of the bleeding or retaining conception products in an incomplete miscarriage when the surgical evacuation was considered necessary.

There were some shortcomings in this study. First, this analysis was performed retrospectively. Second, the surgical evacuation procedure was not limited to the same doctor and the operator’s skills could be different, which may cause variation.

## Conclusion

There was no significant difference in the pregnancy outcomes of subsequent embryo transfer cycle between surgical evacuation patients and no surgical evacuation patients. However, surgical evacuation led to the decrease of EMT, especially when the EMT < 8 mm, which was associated with a lower live birth rate. Unnecessary surgical evacuation should be avoided when possible. However, the miscarriage management should not be unduly conservative when there is the evidence of requisite surgical evacuation.

## Data Availability

The data that support the findings of this study are available from Drum Tower Hospital but restrictions apply to the availability of these data, which were used under license for the current study, and so are not publicly available. Data are however available from the authors upon reasonable request and with permission of Drum Tower Hospital.

## Abbreviations

AFC: Antral follicle count
ART: Assisted reproduction technology
BMI: Body mass index
CI: Confidence interval
D&C: Dilatation and curettage
E2: Oestradiol
EMT: Endometrial thickness
FET: Frozen embryo transfer
FSH: Follicle-stimulating hormone
hCG: Human chorionic gonadotropin
ICSI: Intracytoplasmic sperm injection
IVF-ET: In vitro fertilization and embryo transfer
LH: Luteinizing hormone
OR: Odds ratio
P: Progesterone
